# Choroidal thickness increase after micropulse transscleral cyclophotocoagulation

**Published:** 2018

**Authors:** Ramona Barac, Maria Vuzitas, Florian Balta

**Affiliations:** * ”Carol Davila” University of Medicine and Pharmacy, Bucharest, Romania; ** Retina Clinic, Bucharest, Romania

**Keywords:** transscleral cyclophotocoagulation, choroidal thickness, micropulse, glaucoma surgery

## Abstract

Glaucoma, the affliction that results in optic nerve damage and vision loss, is the main cause of irreversible blindness. The goal of this study was to describe our experience and OCT findings regarding glaucoma patients who underwent MicroPulse Transscleral Cyclophotocoagulation. A variety of glaucoma patients treated with MP-TSCPC were included in our study. LASER settings were 2000mW of 810nm infrared diode micropulse LASER, 31.3% duty cycle and the duration of treatment was between 80-130 s per hemisphere to each eye, at 3 mm of corneoscleral limbus, spearing the nasal and temporal clock hours and also the region with previous filtration surgeries (trabeculectomy). We conducted a prospective study in which twenty-two patients underwent MP-TSCPC under local anaesthesia and they were examined one week, one month, three months, and six months postoperatively. Mean IOP dropped from 35.23 mmHg preoperatively to 17.73mmHg (49.67%) at 1 week follow-up, to 21.81 mmHg (38.09%) at 1 month follow-up, to 22.34 mmHg at 3 months follow-up and to 23.56 mmHg at 6 months follow-up. Four patients (15.8%) underwent a second treatment (at 1 month after the initial treatment) due to insufficient IOP decrease, two of them with success in lowering the IOP postoperatively. By measuring the foveolar choroidal thickness via macular OCT scan, we noticed that all responsive patients had a thicker choroid one week after the laser treatment, with a steady increase of a mean 7.3% that was sustained at one and three months follow-up, while in non-responsive patients, the choroidal thickness remained the same postoperatively, or had a significant decrease. The increase in choroidal thickness in all patients in whom we observed IOP reduction was a significant correlation that supported the mechanism of increased uveoscleral outflow obtained from LASER treatment.

## Introduction

It is well known that glaucoma is the leading cause of irreversible blindness, affecting almost 50 million people worldwide, a prevalence set to more than double in the next 25 years [**1**,**2**]. The economic burden of glaucoma is estimated around 455-969 E/person/year in Europe depending on disease stage [**3**], a number that can be extrapolated to around 6 billion E worldwide in 2040. From a patient perspective, glaucoma is accompanied by an important decrease in quality of life related to visual function and field loss (outdoor mobility and driving, reading, recognizing faces, etc.), ocular surface symptoms and pain [**4**]. 

Glaucoma treatment has two main goals: slowing disease progression and preservation of quality of life [**5**,**6**]. The only proven method to treat glaucoma is the reduction of intraocular pressure [**5**,**7**]. Several multicentric clinical trials showed results that demonstrate the benefit of lowering intraocular pressure in preventing the development and slowing the disease’s progression [**5**,**8**-**11**]. 

Current glaucoma therapies include topical and systemic drugs, LASER surgery (argon laser trabeculoplasty, selective LASER trabeculoplasty, LASER peripheral iridotomy, cycloablation), minimally invasive glaucoma surgeries and traditional glaucoma surgeries [**12**]. Despite using all therapeutic methods to treat this affliction, some cases are still very difficult to manage leading slowly but surely to blindness. 

The new Micropulse Transscleral Cyclophotocoagulation (MPTSCPC) is a non-incisional, noninvasive LASER treatment for glaucoma, which can precede or follow any procedure. This procedure can be repeated as many times as needed and it can be used for simple or complex cases [**13**].

MicroPulse technology controls thermal elevation by “chopping” a continuous-wave beam into an envelope of repetitive short pulses. The MP-TSCPC device revolutionizes cyclophotocoagulation by using MicroPulse technology, where the 31.3% duty cycle signifies that the LASER is off 68.7% of the time, thereby avoiding focal heating and burning of the tissue. The technique of gliding the MP3 device back and forth over 1 hemisphere of the ciliary body results in a slow, steady application of LASER energy [**14**].

## Material and methods

**Fig. 1:**
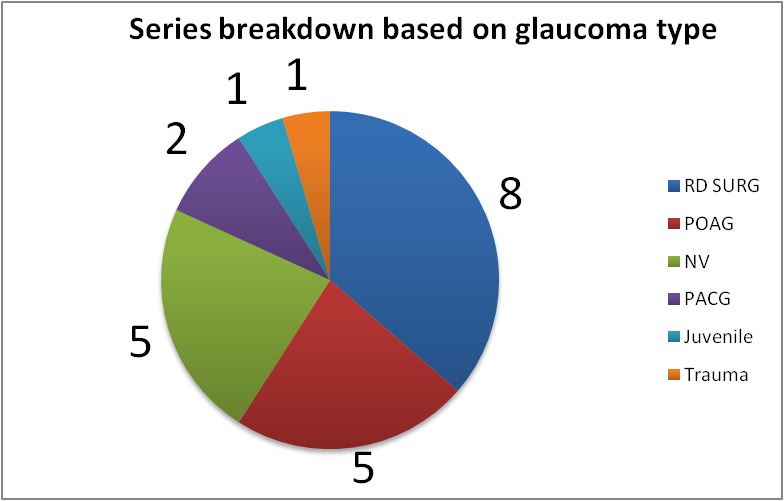
Series breakdown based on glaucoma type

We conducted a prospective study on a series of 22 Caucasian patients with glaucoma (22 eyes) of various etiology (8 patients with secondary glaucoma after retinal detachment surgery and silicon oil removal, 5 patients with primary open-angle glaucoma, 5 patients with neovascular glaucoma, 2 patients with primary angle-closure glaucoma, 1 patient with juvenile glaucoma and 1 patient with secondary posttraumatic glaucoma), aged between 25 and 85 years old (mean age of 50 years old) and predominantly male (17 males and 5 females), as seen in **Fig. 1**. Patients underwent MicroPulse Transscleral Cyclophotocoagulation in Retina Clinic under local anaesthesia between May 2017 and August 2017. LASER settings were 2000mW of 810nm infrared diode micropulse LASER, 31.3% duty cycle and the duration of treatment was between 80 and 130 s per hemisphere to each eye, at 3 mm from the corneoscleral limbus, spearing the nasal and temporal clock hours and also the region with previous filtration surgeries (trabeculectomy). Adjuvant therapy included a short course of postoperative topical NSAIDs. The following parameters were recorded for each patient: age, gender, glaucoma diagnosis and ocular history, preoperative IOP, number of glaucoma medications prior to surgery, preoperative visual acuity (VA) and choroidal thickness (via macular OCT). Intraocular pressure (IOP), pain, medications, visual acuity (VA), macular OCT with foveolar choroidal thickness measurement and complications were recorded at each postoperative visit. After the laser treatment, the surgeon reduced the hypotensive medication considering the rate of IOP decrease. Patients were examined 1 week, 1 month, 3 months, and 6 months postoperatively. 

## Results

**Fig. 2:**
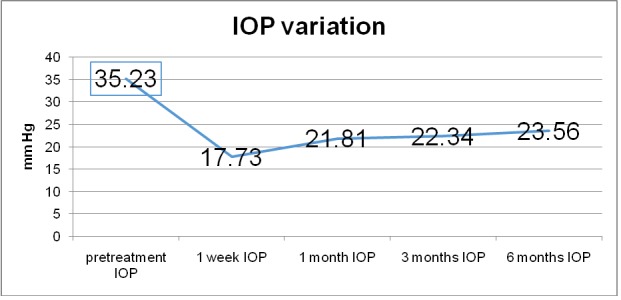
IOP variation before and after MP-TSCPC

Mean IOP dropped from 35.23 mmHg preoperatively to 17.73mmHg at 1 week follow-up, representing a 49.67% decrease, to 21.81 mmHg at 1 month follow-up, representing a 38.09% decrease, to 22.34 mmHg (36.58%) at 3 months follow-up and to 23.56 (33.12%) mmHg at 6 months follow-up as shown in **Fig. 2**.

**Fig. 3:**
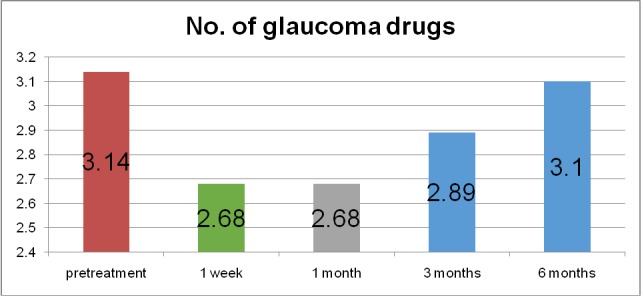
Number of glaucoma drugs before and after MP-TSCPC

**Fig. 4:**
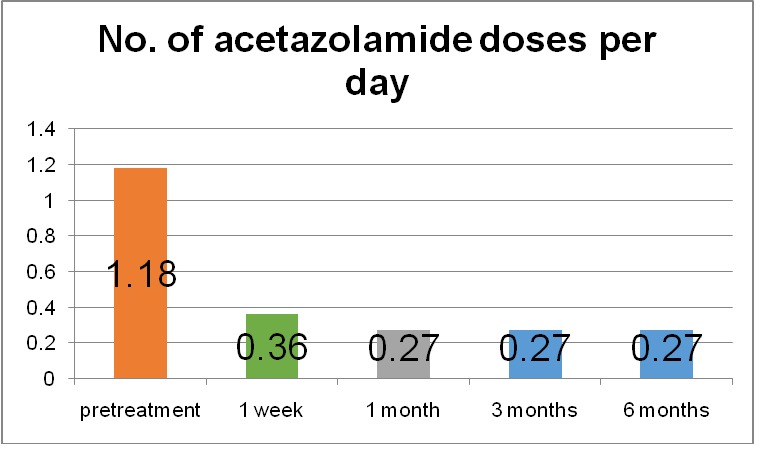
Number of acetazolamide doses per day before and after MP-TSCPC

The average number of glaucoma medications used decreased from 3.14 preoperatively to 2.68 postoperatively at the first two follow-ups. At the last two follow-ups, the number of glaucoma drugs increased at 2.89 at 3 months postoperatively and 3.1 at the 6 months follow-up (**Fig. 3**). Acetazolamide use dropped from 1.18 dose per day to 0.36 dose per day (69.49% reduction) at 1 week follow-up and to 0.27 dose per day (77.11% reduction) at 1 month, 3 months and 6 months follow-up (**Fig. 4**).

Four patients (15.8%) underwent a second treatment (at 1 month after the initial treatment) due to insufficient IOP decrease, two of them with success in lowering the IOP postoperatively. Five patients mentioned blurriness in vision after the laser treatment, with a loss of 1 to 2 lines in VA at 1-week follow-up, one of which presented with herpetic keratitis, but all of them recovered the VA at the 1-month follow-up. Four patients presented with keratic precipitates, for which we added topical steroid (1 drop, 3 times daily for 3 weeks). One patient had a significant decrease in VA due to vitreous haemorrhage and one patient gained one line in VA at one-month follow-up. 

**Fig. 5:**
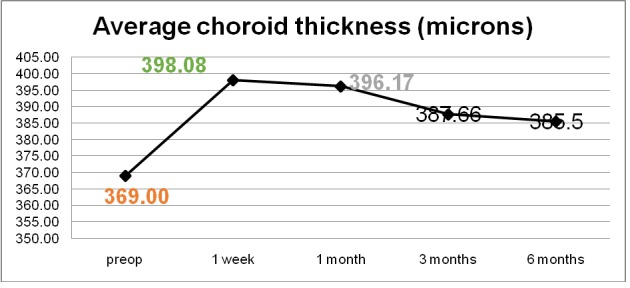
Average choroidal thickness before and after MP-TSCPC

**Fig. 6:**
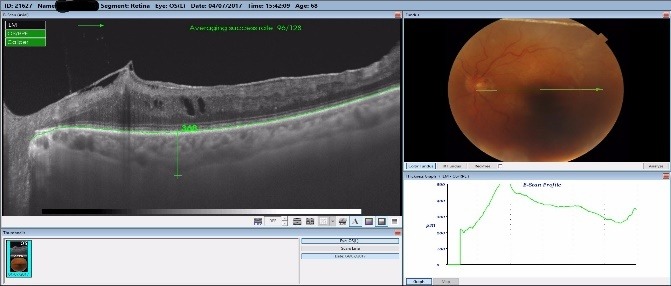
Choroidal thickness preoperatively

**Fig. 7:**
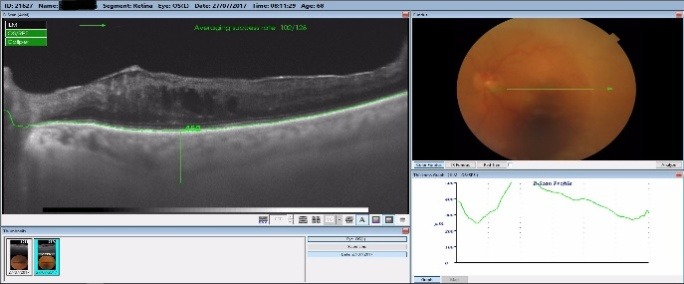
Choroidal thickness 1 week postoperatively

By measuring the foveolar choroidal thickness via macular OCT scan, we noticed that all responsive patients had a thicker choroid one week after the laser treatment, with a steady increase of a mean 7.3% that was sustained at one-month follow-up, as seen in **Fig. 5**-**7**. The choroidal thickness slowly decreased at 3 months and 6 months follow-up, but the value remained higher than preoperatively (**Fig. 5**). All the patients with a good response to the laser treatment showed an increase in choroidal thickness one week postoperatively, and a slight decrease one month postoperatively, but still with a thicker choroid than preoperatively. This might have been related to the increase in the uveoscleral outflow that this laser claimed to do. In some responsive patients, the choroidal thickness increased one month postoperatively, with values greater than before the treatment. 

We observed that in non-responsive patients, the choroidal thickness remained the same postoperatively, or had a decrease. 

**Fig. 8:**
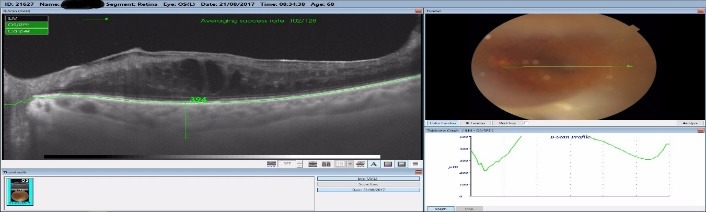
Choroidal thickness 1 month

## Discussion

Our experience with MP-TSCPC showed a 33.12% reduction in IOP at last follow-up, a rate that is similar to other published reports on MP-TSCPC [**15**]. There were no cases of hypotony, phthisis bulbi, or ocular prolonged inflammation. There were no observable side effects from MP-TSCFC and this procedure was well tolerated by patients. In responsive patients, as the choroidal thickness increased, the IOP decreased with the most spectacular result 1 week postoperatively. One of the non-responders had unmodified choroidal thickness at 1-week follow up and one had a significant decrease of 16% and 18% in thickness postoperatively. Two of the patients who failed to respond to the laser treatment had advanced primary open angle glaucoma, one patient had neovascular glaucoma, and type 1 diabetes and the last patient had secondary glaucoma after retinal detachment surgery. It is clear that patients treated with this LASER have a growth in choroidal thickness postoperatively, but it is important to see if there is a correlation between the anatomic choroid type and the LASER treatment outcome, this way, the treatment could become predictable. Although our case series was too small to be statistically significant, patient outcomes strongly suggested that the growth in the choroidal thickness after MP-TSCPC treatment was proof of efficient response. As far as we know, this is the first study to focus on choroid thickness gain in measuring MP-TSCPC treatment effectiveness. We will conduct further studies with an increased number of cases and longer follow-up in order to obtain statistically significant data. 

## Conclusions

Choroidal thickness variation may be the result of the rise in uveoscleral outflow after MP-TSCPC. MP-TSCPC is a safe and effective treatment option for a variety of glaucoma types. Not only can it be used in patients with advanced glaucoma, but also in mild glaucoma cases. MP-TSCPC reduces IOP effectively in the majority of the case series patients with good values even 6 months postoperatively. There were no side effects and patients tolerated the procedure very well. Visual acuity was generally not affected by this procedure. In responsive patients, we noticed a significant growth in choroidal thickness, which was maintained at 6 months follow-up. Non-responsive patients had no choroidal thickness gain postoperatively. 
